# Use of 4-Nitroquinoline 1-Oxide (4NQO) in Dysplastic and Malignant Induction: In Vitro and In Vivo Studies

**DOI:** 10.3390/biomedicines13092223

**Published:** 2025-09-10

**Authors:** Daniela Oliveira Meneses, Brunna da Silva Nobrega Souza, Mateus José Dutra, Isabella Souza Malta, Bruna Oliveira Silva, Isis Moraes Cançado, Nathan Stevan Cezar Conceição, Maria Leticia de Almeida Lança, Luana Marotta Reis de Vasconcellos, Estela Kaminagakura

**Affiliations:** Department of Bioscience and Oral Diagnosis, Institute of Science and Technology, São Paulo State University (UNESP), São José dos Campos 12245-000, São Paulo, Brazil; daniela.meneses@unesp.br (D.O.M.); brunna.nobrega@unesp.br (B.d.S.N.S.); mateusdutra2@hotmail.com (M.J.D.); isabella.malta@unesp.br (I.S.M.); bo.silva@unesp.br (B.O.S.); isis.moraes@unesp.br (I.M.C.); nathan.stevan@unesp.br (N.S.C.C.); leticia.lanaa@unesp.br (M.L.d.A.L.); luana.marotta@unesp.br (L.M.R.d.V.)

**Keywords:** carcinogenesis, 4-nitroquinoline-1-oxide, oral leukoplakia, squamous cell carcinoma of the head and neck, cell line

## Abstract

**Objectives:** Tobacco has been associated with the development of oral leukoplakia (OL) and oral squamous cell carcinoma (OSCC). This study aimed to evaluate the in vitro and in vivo changes caused by carcinogen 4-nitroquinoline 1-oxide (4NQO), simulating smoking conditions. **Materials and Methods:** In the in vitro study, normal keratinocytes were exposed to 1.3 µM and 2.6 µM concentrations of 4NQO to induce dysplastic transformation (H-DISP) and malignant transformation (H-SCC), respectively. The cells were collected and subjected to hematoxylin and eosin (H&E) staining and immunocytochemistry with Ki-67. For the in vivo study, female C57BL/6J mice were divided into a pure control (PC) group and experimental groups exposed to 50 µg/mL (NQ) and 100 µg/mL (CM) of 4NQO in autoclaved drinking water. Each group was euthanized after 8, 12, 16, and 20 weeks of exposure. The tongues were collected, processed, stained with H&E, and analyzed using conventional light microscopy. **Results:** In vitro, significant morphological changes were observed in the H-DISP and H-SCC groups, with a cell proliferation index exceeding 30% in the H-DISP group. In vivo, the CM group showed greater progression to severe dysplasia/carcinoma within a shorter treatment period compared to the NQ group. **Conclusions:** We established critical doses and exposure durations for 4NQO, both in vitro and in vivo, to induce cellular changes and the formation of OL and OSCC, providing a standardized model for studies related to oral carcinogenesis.

## 1. Introduction

Oral leukoplakia (OL) is a predominantly white plaque of uncertain risk that excludes other known diseases or disorders not associated with increased cancer risk [1]. It is the most common oral potentially malignant disorder (OPMD) of the oral cavity [2], with tobacco and alcohol being the primary risk factors for its development [2], different from proliferative verrucous leukoplakia (PVL), which is considered its multifocal form [3]. Histological analysis of OL may reveal simple hyperkeratosis, varying degrees of epithelial dysplasia (ED), or even oral squamous cell carcinoma (OSCC) [4,5,6]. Conventional keratinizing is the most common subtype of OSCC; however, several other variants have been described, including spindle cell squamous cell carcinoma, basaloid squamous cell carcinoma, acantholytic squamous cell carcinoma, adenosquamous carcinoma, papillary squamous cell carcinoma, and lymphoepithelial carcinoma [6].

The relationship between tobacco use and OSCC development is well-established [7]. Tobacco, in both smokable and chewable forms, contains more than 60 identified carcinogenic substances, including polycyclic aromatic hydrocarbons (PAHs) and nitrosamines, which directly damage DNA [8], potentially leading to genetic mutations, the deregulation of cell proliferation, and apoptosis [7].

Understanding the initiation and progression of oral carcinogenesis is crucial for developing effective strategies to prevent the malignant transformation of OL into OSCC. To achieve this, studies must assess the relationship between cigarette consumption (both quantity and duration) and associated cytological and tissue changes. 4-nitroquinoline 1-oxide (4NQO) is a carcinogen used to induce oral carcinogenesis in mice, exhibiting up to 94% mutational similarity, as well as histological and progression similarities, to OSCC in humans [9,10]. Therefore, our objective was to evaluate, both in vitro and in vivo, the cytological and architectural changes caused by 4NQO at different concentrations and exposure times, simulating smoking conditions and establishing a versatile platform for future studies.

## 2. Materials and Methods

### 2.1. In Vitro Study

#### 2.1.1. Selection of the Cell Line

HaCat cell line (American Type Culture Collection, Manassas, VA, USA) of immortalized human keratinocyte was cultured in high-glucose DMEM (Nova Biotecnologia, Cotia, SP, Brazil), supplemented with 10% fetal bovine serum (Nova Biotecnologia, Cotia, SP, Brazil) and 1% antibiotic (penicillin–streptomycin amphotericin, Nova Biotecnologia, Cotia, SP, Brazil) at 37 °C and 5% CO_2_ [11].

#### 2.1.2. Induction of Malignancy and Cellular Dysplasia

After reaching 90% confluence, 4NQO (Sigma-Aldrich, St. Louis, MO, USA) was diluted in culture medium at a concentration of 2.6 µM for malignant transformation (H-SCC) and at 1.3 µM for dysplastic transformation (H-DISP). The diluted solution was added for 1 h 30 min, once. After this period, the culture medium containing 4NQO was removed, and a fresh medium was added for cell recovery. These protocols were adapted from Crane et al. [12].

The culture medium was replaced every two days until the cells reached confluence for collection for hematoxylin and eosin (H&E) staining and immunocytochemistry reactions. The cells were collected using a sterile cytobrush (PQF, Ciudad de México, Mexico) and smeared onto glass slides (Starfrost, Knittel Glass, Braunschweig, Germany), which were then fixed in 95% alcohol (Quimesp Química, Guarulhos, SP, Brazil) [13].

#### 2.1.3. H&E Staining

The slides were washed with distilled water for 1 min, stained with H&E, and analyzed under conventional light microscopy. Cellular changes such as an increased nucleus/cytoplasm ratio, nuclear hyperchromatism, an increased number of mitoses, aberrant mitotic figures, and architectural changes were analyzed [6]. The classification was performed by two independent researchers (D.O.M. and M.J.D.), and in cases of disagreement, a third researcher was consulted (E.K.).

#### 2.1.4. Immunocytochemistry

The silanized slides (Starfrost, Knittel Glass, Braunschweig, Germany) containing the fixed HaCat, H-DISP, and H-SCC cells in 95% alcohol underwent decreasing concentrations of alcohol baths for hydration. Antigen retrieval was performed by immersion in ethylenediaminetetraacetic acid (EDTA) solution (1 mM, pH 8.0) and incubated for 40 min in a water bath at 97 °C. Endogenous peroxidase blocking was performed with three baths of 20% hydrogen peroxide (Merck, Rio de Janeiro, RJ, Brazil) and methanol (Merck, Rio de Janeiro, RJ, Brazil) in a 1:1 ratio, with each bath lasting 10 min. Bovine serum albumin (BSA) (Sigma-Aldrich, St. Louis, MO, USA) was then used for blocking at a concentration of 0.1 g for 1 h. Ki-67 antibody incubation (Novus Biologicals, Centennial, CO, USA) was performed at a concentration of 1:1000 overnight. Signal amplification was performed with horseradish peroxidase (HRP) (Dako Cytomation, Glostrup, Denmark) for 30 min, staining was performed with diaminobenzidine (DAB) (Dako Cytomation, Glostrup, Denmark) for 3 min, and counterstaining with Harris’ hematoxylin was performed for 1 min. The slides were dehydrated with increasing concentrations of alcohol, and finally treated with two xylene baths before being mounted in a permanent medium. OSCC and fibrous hyperplasia cases were used as positive and negative controls in all reactions for Ki-67 [9,14,15].

### 2.2. In Vivo Study

#### 2.2.1. Ethical Considerations

The handling of mice was conducted in accordance with the Ethical Principles for Animal Experimentation adopted by the National Council for the Control of Animal Experimentation (CONCEA) (ARRIVE Checklist), and approved by the Research Ethics Committee of the Institute of Science and Technology of São Paulo State University under protocol number 02/2021.

#### 2.2.2. Sample

Forty-five female C57BL/6J mice aged 4–6 weeks, weighing approximately 16–18 g, were used. The animals were obtained from the Animal Core Facility of UNESP. They had ad libitum access to water and feed. The environment was carefully monitored to maintain a temperature of around 20 °C and a humidity of 55%.

#### 2.2.3. Group Division

**Figure 1:**
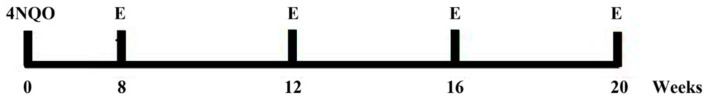
Euthanasia periods (E), performed after the start of 4NQO administration.

After 30 days of acclimatization to the environment, with daily cycles alternating between 12 h of light and 12 h of darkness, the experiments were initiated. The animals were divided into two test groups: the NQ group, composed of 20 animals that received 4NQO at a concentration of 50 µg/mL in their drinking water, and the CM group, composed of 20 animals that received 4NQO at a concentration of 100 µg/mL in their drinking water. The test groups were evaluated in four periods: 8, 12, 16, and 20 weeks (Figure 1), thus, 5 animals from each group were euthanized in these different time periods. The pure control group (PC) was composed of 5 animals that were euthanized in the eighth week, adapted from Dutra et al. (2024) [11].

#### 2.2.4. Induction of OL and OSCC

The induction of OL and OSCC was performed by exposing the animals to 4NQO (Sigma-Aldrich, St. Louis, MO, USA), which was diluted in propylene glycol at a concentration of 4 mg/mL (stock solution). The stock solution was diluted in autoclaved water to reach final concentrations of 50 µg/mL and 100 µg/mL, being prepared exclusively during reservoir changes performed twice a week. The 4NQO-containing water was protected from light [11,16].

#### 2.2.5. Sample Collection, Macroscopic Analysis, and Preparation

After 8, 12, 16, and 20 weeks of exposure to 4NQO, the animals were euthanized (Figure 1), in a total of 5 animals in different time periods. The tongues were collected and macroscopically analyzed for clinical changes. They were then longitudinally hemisectioned into two parts and fixed in 4% formaldehyde (Sigma-Aldrich, St. Louis, MO, USA). The obtained samples were processed, embedded in paraffin, subjected to routine histological techniques, stained with H&E, and then analyzed under conventional light microscopy.

#### 2.2.6. Histological Analysis

The obtained slides were evaluated by two independent researchers (M.J.D. and B.d.S.N.S.), and a third researcher (E.K.) was consulted in cases of discrepancy. Tissue changes were classified according to the World Health Organization (WHO) classification into mild dysplasia, severe dysplasia, and OSCC [6].

#### 2.2.7. Statistical Analysis

The analysis under conventional light microscopy (Zeiss−Axioskop 40) was performed blindly. The KAPPA test was applied to assess the level of agreement between the evaluators of the classifications, resulting in 70.6%. In cases of disagreement, a discussion was held to determine the final qualitative results.

## 3. Results

### 3.1. In Vitro Study

#### 3.1.1. Microscopic Analysis of Cultured Cells

**Figure 2:**
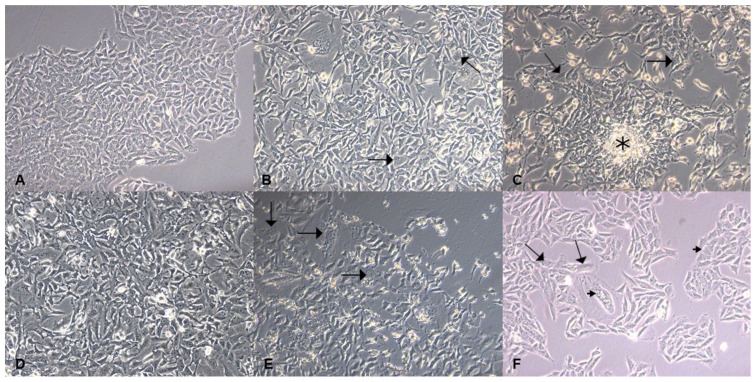
Microscopic analysis of cultured cells, cytoplasmic morphological alterations are pointed by long arrows and nuclear alterations are shown by short arrows; asterisks represent growth-forming nodules. (**A**) Control HaCaT cells (40× magnification); (**B**) H-DISP cells 7 days after treatment (40× magnification); (**C**) H-DISP cells 2 weeks after treatment (40× magnification); (**D**) control HaCaT cells (40× magnification); (**E**) H-SCC cells 7 days after treatment (40× magnification); (**F**) H-SCC cells 2 weeks after treatment (40× magnification).

Cells that did not receive 4NQO did not show morphological or adhesion changes (Figure 2A,D). In the cells exposed to a 1.3 µM concentration of 4NQO for the induction of cellular dysplasia, 7 days after single exposure to the carcinogen, the H-DISP group exhibited subtle alterations in cellular architecture (shown by the arrows), which were noted during the cultivation of these cells (Figure 2B). After 2 weeks, the cells regained their adhesion capacity, and the observed characteristics included a spread morphology, enlarged nuclei (shown by the short arrows), and growth-forming nodules (shown by the asterisk) that characterize cell overlap during cultivation (Figure 2C).

In the cells exposed to a 2.6 µM concentration of 4-NQO for the induction of malignant transformation, 7 days after single exposure to the carcinogen 4-NQO, few viable cells remained. The surviving cells exhibited morphological alterations (shown by the arrows) (Figure 2E). Two weeks after single exposure to 4NQO, the cells demonstrated improved adhesion and proliferation capacity, while displaying nuclear (shown by the long arrows) and cytoplasmic atypia (shown by the short arrows) during cell culture (Figure 2F).

#### 3.1.2. Hematoxylin and Eosin Analysis

**Figure 3:**
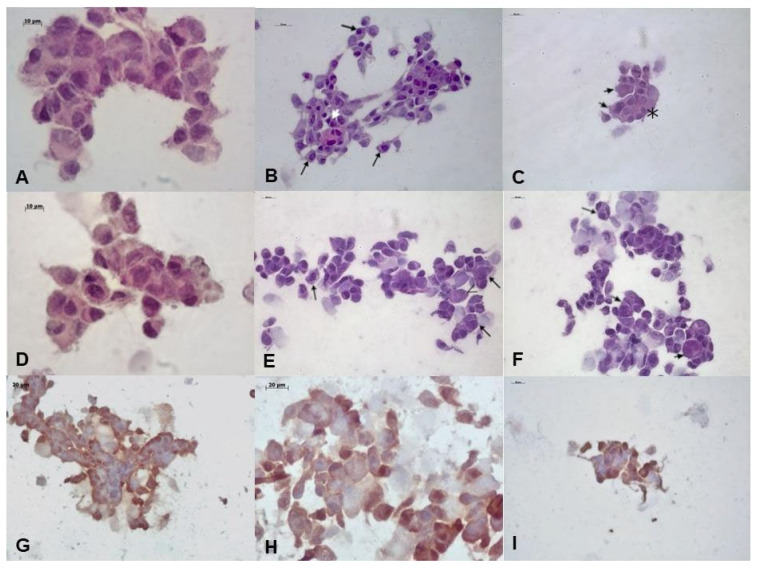
Microscopic analysis of cultured cells. (**A**) Control HaCaT cells (HE, 63× magnification); (**B**) Presents increased cytoplasmatic and nuclear sizes (long arrows); (**B**,**C**) H-DISP cells showing slight pleomorphism (bizarre cells pointed by short arrows) and altered nucleus/cytoplasm ratio (asterisks) (HE, 40× magnification); (**D**) control HaCaT cells (HE, 63× magnification); (**E**,**F**) H-SCC cells showing pleomorphism (short arrows), atypia, altered nucleus/cytoplasm ratio (long arrows), and atypical mitotic figures (HE, 40× magnification); (**G**) control HaCaT cells (Ki-67, 100× magnification); (**H**) H-DISP cells (Ki-67, 63× magnification); (**I**) H-SCC cells (Ki-67, 40× magnification). The arrows are in different colors only for better visualization.

Cells that received no dose of 4NQO did not show hyperchromatism and architectural changes (Figure 3A,D). In the H-DISP group, conventional light microscopy revealed significant morphological changes, such as increased cytoplasmic and nuclear size (shown by the long arrows), increased number of nucleoli, binucleated cells (shown by the short arrows), and growth-forming nodules that characterize cell overlap (shown by the asterisk) (Figure 3B,C).

In the H-SCC group, conventional light microscopy also revealed significant morphological changes, such as increased cytoplasmic and nuclear size (shown by the long arrows), increased number of nucleoli, and binucleated and bizarre cells (shown by the short arrows). However, few cells survived (Figure 3E,F).

#### 3.1.3. Immunocytochemistry Analysis

The cell proliferation index, investigated through nuclear Ki-67 staining, was low in HaCat cells (Figure 3G), and positive in >30% of the cells in the H-DISP group (Figure 3H). Due to the scarcity of cells obtained, it was not possible to quantify Ki-67 positivity in the H-SCC group (Figure 3I).

### 3.2. In Vivo Study

#### 3.2.1. Macroscopic Analysis

**Figure 4:**
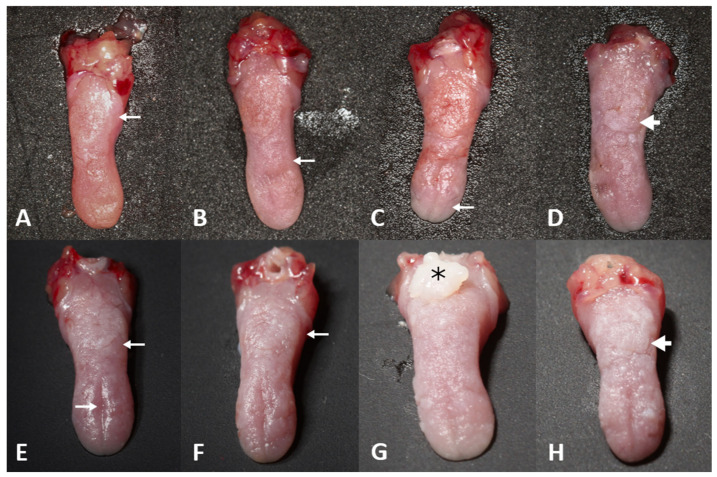
Representative macroscopic photographs of the dorsal tongue of each group. (**A**) NQ8; (**B**) NQ12; (**C**) NQ16; (**D**) NQ20; (**E**) CM8; (**F**) CM12; (**G**) CM16; (**H**) CM20. Thin arrows represent depapillation areas, thick arrows represent verrucous surfaces, and asterisks represent the nodules.

Depapillation of the dorsal tongue (shown by the thin arrow) was observed in all groups (NQ and CM, 8, 12, 16, and 20 weeks), (Figure 4A–C,E,F). At 16 weeks, a sessile, whitish, verrucous-surfaced nodule (shown by the asterisk) was observed in the CM group (Figure 4G). At 20 weeks, thick white plaques with ill-defined borders and verrucous surfaces (shown by the thick arrows) were observed in some tongue areas in the NQ and CM groups (Figure 4D,H).

#### 3.2.2. Histological Analysis

The microscopic grades are summarized in Table 1.

**Figure 5:**
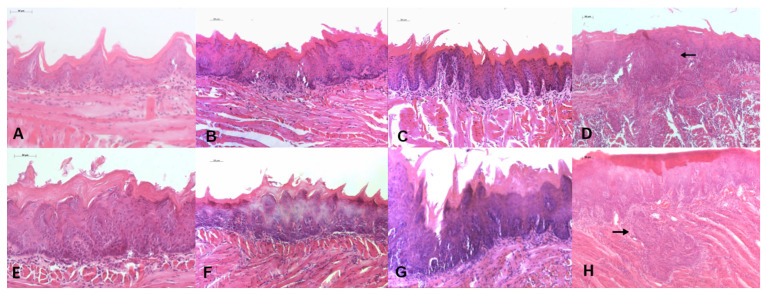
Representative photomicrographs of histological slides from each group. (**A**) NQ8 (HE, 200× magnification); (**B**) NQ12 (HE, 200× magnification); (**C**) NQ16 (HE, 200× magnification); (**D**) NQ20 (HE, 100× magnification); (**E**) CM8 (HE, 200× magnification); (**F**) CM12 (HE, 200× magnification); (**G**) CM16 (HE, 200× magnification); (**H**) CM20 (HE, 100× magnification). The arrows indicate areas of carcinoma.

In the NQ8 group, four animals had mild to moderate dysplasia (80%) (Figure 5A). In the NQ12 and NQ16 groups, mild to moderate dysplasia was found in all evaluated animals (100%) (Figure 5B,C). In the NQ20 group, carcinoma (shown by the arrows) were found in all evaluated animals (100%) (Figure 5D).

In the CM8 group, mild to moderate dysplasia was observed in all evaluated animals (100%) (Figure 5E). In CM12, mild/moderate dysplasia was found in 80% of the animals (Figure 5F). In CM16, mild to moderate dysplasia was found in 40% of the animals, and severe dysplasia and carcinoma were found in the other 60% (Figure 5G). In the CM20 group, severe dysplasia and carcinoma (shown by the arrow) were found in all evaluated animals (100%) (Figure 5H).

## 4. Discussion

The development of experimental carcinogenesis models is essential for advancing the understanding of the biological mechanisms of OSCC and improving prevention, diagnosis, and treatment strategies. Experimental models allow investigation of the signaling pathways involved in malignant transformation, the development and validation of biomarkers, and the evaluation of new therapies [11].

4NQO and 7,12-dimethylbenz[a]anthracene (DMBA) are widely used as carcinogenic agents in experimental models of oral carcinogenesis [8]. DMBA is a polycyclic aromatic hydrocarbon that is metabolized into 7,12-DMBA-3,4-oxide, which is subsequently converted into 7,12-DMBA-3,4-diol by epoxide hydrolase and then into 7,12-DMBA-3,4-diol-1,2-epoxide by cytochrome P450 enzymes [17,18]. The formation of this electrophilic metabolite is crucial for the toxicity of DMBA, leading to DNA adduct formation, mutations, and the deregulation of gene expression and intracellular signaling [18].

4-NQO, a synthetic molecule derived from quinoline composed of two polar groups, is classified as a genotoxic substance that reacts with DNA, resulting in the formation of adducts in genetic material [19]. Upon metabolism, it transforms into 4-hydroxyaminoquinoline 1-oxide (4HAQO) and 4-aminoquinoline 1-oxide (4AQO), with only 4HAQO considered carcinogenic. After acetylation, it reacts with DNA, covalently binding to deoxyguanosine residues at C8 or N2 and deoxyadenosine at N6 [20,21], causing intracellular oxidative stress, DNA damage, and promoting TP53 mutations, which initiate and drive the progression of oral cancer [8,10]. In mice, 4NQO mimics the tobacco-related carcinogenic signature, showing approximately 94% mutational similarity to human OSCC, providing a comparable histological and molecular landscape [8,10,22]. In contrast, DMBA exhibits only 39.7% similarity to tobacco-induced events in humans [10].

In this study, 4NQO was selected due to its solubility in water, allowing for continuous and uniform administration. DMBA requires topical application, necessitating frequent handling and increasing the risk of carcinogen exposure [23,24]. Protocols using this carcinogen involve multiple topical applications of a 0.5% or 0.25% DMBA solution in mineral oil or acetone via brush [23,24]. Additionally, DMBA or its solvent may cause local irritation, triggering an inflammatory response, necrosis, and desquamation, complicating subsequent histopathological analysis [8].

Macroscopic analysis revealed depapillation of the dorsal tongue in all groups. In the CM16 group, a nodule formed, whereas in the CM20 and NQ20 groups, white plaques were observed, consistent with Hawkins et al., who applied 4NQO (5 mg/mL in propylene glycol) topically three times per week for up to 16 weeks [25]. 4NQO-induced lesions in mice tend to develop more rapidly on the tongue [25] and can manifest in various forms, including white plaques followed by reddened, erosive, ulcerated areas and nodules [26].

We observed that the 50 µg/mL dose over 8 weeks resulted in mild to moderate dysplasia in 80% of the animals, making it a relevant dose for studying OL. After 12 weeks, animals receiving 50 µg/mL and 100 µg/mL presented varying degrees of OSCC and dysplasia. Ribeiro and Salvadori [27] administered 4NQO at 50 µg/mL in drinking water to rats and found mild to moderate dysplasia after 12 weeks, with moderate to severe dysplasia and OSCC after 20 weeks. However, Hasina et al. [28], reported that 50% of animals developed OSCC under the same concentration and exposure time.

For the 100 µg/mL concentration, our results showed a progressive development of dysplastic lesions over time, similar to the findings of Tan et al. [9], where 100% of the mice developed mild dysplasia between 3 and 8 weeks, 60% exhibited moderate dysplasia, and 40% presented severe dysplasia between 9 and 12 weeks. Between weeks 13 and 16, all animals showed moderate dysplasia, while between weeks 17 and 20, 80% exhibited severe dysplasia. However, it is important to closely monitor the animals’ behavior, as the 100 µg/mL dose can accelerate lesion development and worsen health conditions, potentially leading to premature death.

Our findings demonstrated that the administration of 50 µg/mL and 100 µg/mL of 4NQO in drinking water for 12 weeks resulted in the development of hyperplastic, dysplastic, and OSCC lesions. Similarly, exposure to 4NQO at concentrations of 20 µg/mL, 50 µg/mL, and 100 µg/mL for 8 to 16 weeks has been reported to induce these lesions [29].

In vitro, we tested 1.3 µM and 2.6 µM concentrations of 4NQO to induce dysplastic and malignant transformation in normal keratinocyte cell lines, adapting the method from Crane et al. [12]. After exposure to the carcinogen, we observed a reduction in the number of viable cells, regardless of the doses used. Kartasova et al. [30] tested a 4NQO concentration of 0.5 mM for 4 h and found that only 5 to 10% of cells survived. There is a reduction in cell viability as the 4NQO concentration increases [31]. DNA repair occurs within 24 h after 4NQO removal in cultured cells [31], promoting cell recovery and replication, as observed in our study, where the H-DISP cell population increased after a few days.

Morphological alterations were more evident at a concentration of 2.6 µM, where a lack of cell anchorage was observed, making it difficult to accumulate some cells that received this concentration for certain experiments. However, it is worth noting that the loss of anchorage is indicative of malignant transformation [32], and was a significant characteristic observed in our study.

4-NQO can also be used to study the expression of cellular phenotypes in vitro in oral carcinogenesis, particularly when combined with in vivo studies [12]. The study was limited by the small number of cells available for proliferation analyses and by the use of a single cell line for malignant transformation. Future research should include additional cell lines and carcinogenesis markers to strengthen the findings. Another limitation was the inability to assess the tumorigenic potential of these cells in animal models after 4NQO exposure.

## 5. Conclusions

A total of 100 µg/mL of 4NQO accelerates lesion progression, reducing the time required for tumor induction and minimizing the prolonged exposure of animals to the carcinogen. However, strict monitoring is necessary to prevent premature animal death. For in vitro studies, 4NQO is suitable for inducing dysplastic and malignant transformation in the HaCat cell line at doses of 1.3 µM and 2.6 µM, respectively.

## Figures and Tables

**Table 1:** Quantitative data of histological findings of the tongues, divided by experimental groups.

Weeks	Classification	NQ *n* (%)	CM *n* (%)
8	Without dysplasia	1 (20)	-
	Mild/moderate dysplasia	4 (80)	5 (100)
	Severe dysplasia/carcinoma	-	-
12	Without dysplasia	-	-
	Mild/moderate dysplasia	5 (100)	4 (80)
	Severe dysplasia/carcinoma	-	1 (20)
16	Without dysplasia	-	-
	Mild/moderate dysplasia	5 (100)	2 (40)
	Severe dysplasia/carcinoma	-	3 (60)
20	Without dysplasia	-	-
	Mild/moderate dysplasia	-	-
	Severe dysplasia/carcinoma	5 (100)	5 (100)

No dysplasia was found in the histological sections (100%) of the pure control (PC). The NQ group received 4NQO at a concentration of 50 µg/mL, and the CM group received the maximum concentration in our study, of 100 µg/mL, both offered in their drinking water.

## Data Availability

The raw data supporting the conclusions of this article will be made available by the authors on request.
